# Decreased Functional Connectivity of Insular Cortex in Drug Naïve First Episode Schizophrenia: In Relation to Symptom Severity

**DOI:** 10.1371/journal.pone.0167242

**Published:** 2017-01-20

**Authors:** Lijuan Pang, David Kennedy, Qinling Wei, Luxian Lv, Jinsong Gao, Hong Li, Meina Quan, Xue Li, Yongfeng Yang, Xiaoduo Fan, Xueqin Song

**Affiliations:** 1 The First Affiliated Hospital of Zhengzhou University, Zhengzhou, China; 2 Department of Psychiatry, University of Massachusetts Medical School, Worcester, Massachusetts, United States of America; 3 Department of Psychiatry, the Third Affiliated Hospital of Sun Yat-Sen University, Guangzhou, China; 4 Henan Province Biological Psychiatry Key Laboratory, Xinxiang Medical University, Xinxiang, China; 5 Henan Province Mental Hospital, the Second Affiliated Hospital of Xinxiang Medical University, Xinxiang, China; University of Texas at Austin, UNITED STATES

## Abstract

**Background:**

This study was to examine the insular cortical functional connectivity in drug naïve patients with first episode schizophrenia and to explore the relationship between the connectivity and the severity of clinical symptoms.

**Methods:**

Thirty-seven drug naïve patients with schizophrenia and 25 healthy controls were enrolled in this study. A seed-based approach was used to analyze the resting-state functional imaging data. Insular cortical connectivity maps were bilaterally extracted for group comparison and validated by voxel-based morphometry (VBM) analysis. Clinical symptoms were measured using the Positive and Negative Syndrome Scale (PANSS).

**Results:**

There were significant reductions in the right insular cortical connectivity with the Heschl’s gyrus, anterior cingulate cortex (ACC), and caudate (*p*’s<0.001) in the patient group compared with the healthy control (HC) group. Reduced right insular cortical connectivity with the Heschl’s gyrus was further confirmed in the VBM analysis (FDR corrected *p*<0.05). Within the patient group, there was a significant positive relationship between the right insula-Heschl’s connectivity and PANSS general psychopathology scores (r = 0.384, *p* = 0.019).

**Conclusion:**

Reduced insula-Heschl’s functional connectivity is present in drug naïve patients with first episode schizophrenia, which might be related to the manifestation of clinical symptoms.

## 1. Introduction

Schizophrenia, a severe psychiatric disorder characterized by positive and negative symptoms and cognitive deficits[1], has long been hypothesized as a disorder of brain connectivity [2]. Disrupted brain development can lead to abnormal neural connectivity or network, which may cause abnormal information processing and integration, and clinical symptoms including psychosis[3–5]. It has been well established that the insula cortex is an anatomical gateway between the visual, olfactory, auditory, somatosensory cortices and the limbic structures. Secluded deep within the lateral sulcus of the human brain, the insular cortex is part of an extended network of neuronal pathways connecting to the anterior cingulate cortex, temporal lobe, parietal lobe, hippocampus, amygdala, olfactory cortex, and thalamus[6]. The ventromedial prefrontal cortex, posterior cingulate cortex, bilateral inferior parietal cortex and middle temporal lobe are parts of default mode network (DMN). The central executive network (CEN) consists of mainly the dorsolateral prefrontal cortex and posterior parietal cortex. The salience network (SN) includes primarily the anterior insular cortex and dorsal anterior cingulate cortex[3,7]. Apparentlythe insular cortex functions as a multimodal sensory integration region. In addition, the insular cortex plays an important role in emotion processing including interoceptive awareness, anticipation, evaluation of emotional stimuli, self-awareness[7], episodic memory[8], executive function [9], attention[10], and saliency processing[11].

Recently, the insula cortex has attracted significant attention in schizophrenia research[12]. The aberrant functional and structural alterations in insular cortex have been frequently reported in schizophrenia including reduced gray matter volume, thickness, and surface area[13], decreased white matter integrity (measured by fractional anisotropy or mean diffusivity)[14], and altered functional activity under various tasks[15] or during resting state[16,17]. Changes in the SN may be one of the most important findings among all insular cortex related networks in schizophrenia[7]. Recently, the anterior insular cortex within the SN has been demonstrated to be crucial to modulate DMN /CEN interactions in patients with schizophrenia[18]. Manoliu et al. also found that the dependence of CEN and DMN interactions on SN’s right anterior insular activity is altered in patients with schizophrenia during acute psychosis[17] or psychotic remission [16]. Furthermore, insular cortical dysfunction might be associated with core symptoms of schizophrenia. Aberrant salience network activity in the insular and cingulate cortices has been implicated in the development of positive symptoms of schizophrenia such as delusions and hallucinations due to an inappropriate assignment of salience to stimuli that would normally be considered irrelevant[3,7,19,20]. Patients with schizophrenia are usually suffer from impaired insight; both awareness and mental state attribution, two core components of insight, are associated with the function of insular cortex[20].

Previous studies have provided compelling evidence supporting the critical role of insular cortex in schizophrenia. However, most studies examining the functional connectivity between insular cortex and other brain areas were in schizophrenia patients treated with antipsychotics. The potential confounding effect of antipsychotic treatment on the brain functional connectivity has been well established[21,22]. The purpose of the present study was to examine the insular cortical functional connectivity and its relationship with the severity of clinical symptoms in drug naïve, first episode schizophrenic patients.

## 2. Methods

### 2.1. Subjects

The study was approved by the Ethics Committee of the First Affiliated Hospital of Zhengzhou University. All subjects provided written informed consent to participate in the study. Subjects were recruited from the consecutive admissions to the inpatient unit between November 2011 and December 2012. Inclusion criteria included: 1) diagnosis of schizophrenia according to the criteria of DSM-IV; 2) 18–45 years old; 3) in the age rangenever treated with antipsychotic medications or other psychotropics. The diagnosis of schizophrenia was confirmed by a research psychiatrist (X.S.) using the Structured Clinical Interview for DSM-IV Axis I Disorders (SCID-IV). Exclusion criteria were: 1) history of alcohol or other substance use; 2) history of brain injury; 3) any ongoing significant medical conditions. Healthy control subjects were recruited from the local community through advertisements. The same research psychiatrist (X.S.) conducted a comprehensive clinical interview to rule out any psychiatric conditions in healthy controls. A complete medical history, physical examination, and routine laboratory tests were obtained from all subjects to rule out possible medical conditions. All subjects were right-handed.

### 2.2. Clinical symptom measurement

Symptoms of schizophrenia were assessed for all patients using the Positive and Negative Syndrome Scale (PANSS), which includes 3 subscales: positive symptoms, negative symptoms and general psychopathology[1,23]. The PANSS was administered by the same rater (J.G.) throughout the study.

### 2.3. Image acquisition

On the same day after clinical assessment and before taking any antipsychotic or other psychotropic medications, all participants were scanned on a 3.0 Tesla Scanner (Signa HDxt 3T GEHCGEHC) at the Magnetic Resonance Center of the First Affiliated Hospital of Zhengzhou University. A 6 minute ‘resting-state’ functional MRI scan was obtained, comprising of 180 time points of whole-brain functional (EPI) volumes (TR = 2000 ms; TE = 30ms; flip angle = 90; 34 contiguous AC–PC aligned axial slices; matrix = 64×64; FOV = 22 cm; acquisition voxel size = 3.4mm×3.4mm×4 mm). During this scan that acquired all neuroimaging data, participants were instructed to rest without moving. The wakefulness of the subject was verified by self-report at the end of the scan. T1-weighted Spoiled Gradient Echo (SPGR) images were also collected for the purposes of anatomical localization. The acquisition protocol included the following pulse sequence and parameters: repetition time (TR) = 12ms, echo time (TE) = 4.5ms, inversion time (TI) = 1100ms, flip angle 7°, field of view (FOV) = 256×256×220 mm^3^, matrix size 256×256, slice thickness 1mm contiguous, and scan time 17 min.

### 2.4. Image processing

Imaging data were coded and catalogued before being transferred to the Psychotic Disorders Program of the University of Massachusetts Medical School (UMMS) for blinded analysis. AFNI [24] was used for image preprocessing (http://www.afni.nimh.gov/afni), performing slice timing correction for interleaved acquisition (using Fourier interpolation), motion correction (by aligning each volume to a “base” image [middle volume] using Fourier interpolation) and de-spiking (detection and reduction of extreme time series outliers using a hyperbolic tangent function). All other data processing was carried out using FMRIB Software Library (FSL) (http://www.fmrib.ox.ac.uk), including spatial smoothing (FWHM = 6 mm), mean-based intensity normalization of all volumes, temporal bandpass filtering (highpass temporal filtering: Gaussian-weighted least-squares straight line fitting, with sigma = 100.0 s; Gaussian lowpass temporal filtering HWHM 2.8 s), and pre-whitening. Each individual’s time series were spatially normalized by registration to the MNI152 (Montreal Neurological Institute) template with 2mm^3^ resolution, using a 12 degree of freedom affine transformation. Nine nuisance covariates (time series for global signal intensity, white matter, cerebrospinal fluid, and six motion parameters) were regressed out of the data to minimize the contributions of artifactual physiological signals (e.g., cardiac and respiratory cycles) using the general linear model implemented in FSL program FEAT. The FreeSurfer software (http://surfer.nmr.mgh.harvard.edu/) was used to segment T1-weighted SPGR images into cortical and subcortical gray and white matter regions, as well as total intracranial volume, for each subject[24].

Functional connectivity was examined using a seed-based approach[25]. The left and right insular seed regions were generated using the Harvard-Oxford atlas [26], co-localized to the MNI space used in this study. For each participant, we calculated the mean time series of each seed by averaging across all voxels within the seed. A voxel-wise correlation map was generated to each of these seed-based time course reference signals. This correlation map was r-to-Z transformed in order to generate a standardized representation. Each of the remaining cortical and subcortical gray matter regions of the Harvard-Oxford atlas were then used as a mask to determine the average resting state correlation (Z-score) between the insular cortex and each ipsilateral gray matter region in each subject.

### 2.5. Statistical analysis

The data were analyzed using SPSS 20.0 (SPSS Inc., Chicago, IL). Demographic and clinical characteristics were reported using descriptive statistics. The Shapiro-Wilk test was used to check the normality of the data. Group comparisons were performed using the independent samples t-test for continuous variables and Chi-square or Fisher’s exact test for categorical variables.

Connectivity values (Z-scores) of the insular cortex with ipsilateral cortical ROIs were extracted for group comparison, using the independent samples t-test. Bonferroni corrections considering the number of ROIs were used to define the significance level: *p*<0.05/48 = 0.001. Insula connectivity maps (Z-score maps) were subjected to voxel-based morphometry (VBM) analysis [27]. To further explore the regions that show group difference in insular connectivity. A false discovery rate (FDR) corrected *p*<0.05 was used to define the overall significant level.

Peasson correlation analysis was used to examine the relationship beteween insular connectivity and clinical symptoms as measured by the PANSS; the significance level was set at *p*<0.05.

## 3. Results

The original study sample included 46 patients with schizophrenia (SZ) and 30 healthy control (HC) subjects. One SZ and 2 HC individuals were excluded due to the poor quality of their T1 scans; one SZ and 1 HC individuals were excluded due to the poor quality (missing time series) of the resting-state scans; Seven SZ and 2 HC individuals were excluded because the imaging values were defined as outliers using the criteria of Mean±2SD. The final study sample used for data analysis was thus composed of 37 SZ subjects and 25 HC subjects.

Table 1 shows demographic and clinical characteristics of the study sample. There were no significant differences between the patient group and the HC group in age, gender, education, and intracranial volume (ICV) (*p*’s > 0.05).

**Table 1:** Demographic and clinical characteristics of the study sample.

Characteristics	HC a (N = 25) (Mean±SD)	SZ b (N = 37) (Mean±SD)	t	*P*
Age(years)	23.16±5.41	22.14±5.02	1.312	0.195
Education(years)	13.52±3.77	12.81±2.12	0.855	0.399
Gender (Male/Female)	15/10	24/13		0.791
ICVc (cm^3^)	1623.47±141.11	1669.23±162.50	-1.145	0.257
Age of onset (years)	N/A	21.7 +3.9		
Duration untreated psychosis (months)	N/A	8.31±7.49		
PANSS-positive	N/A	21.22±2.96		
PANSS-negative	N/A	17.95±2.33		
PANSS-general	N/A	38.32±2.93		
PANSS-total	N/A	77.43±4.20		

HC: healthy control group.

SZ: schizophrenia patient group.

ICV: intracranial volume

PANSS: the Positive and Negative Syndrome Scale, including positive symptoms, negative symptoms, general psychopathology subscales and total scores.

### 3.1. ROI-based analysis

**Fig 1:**
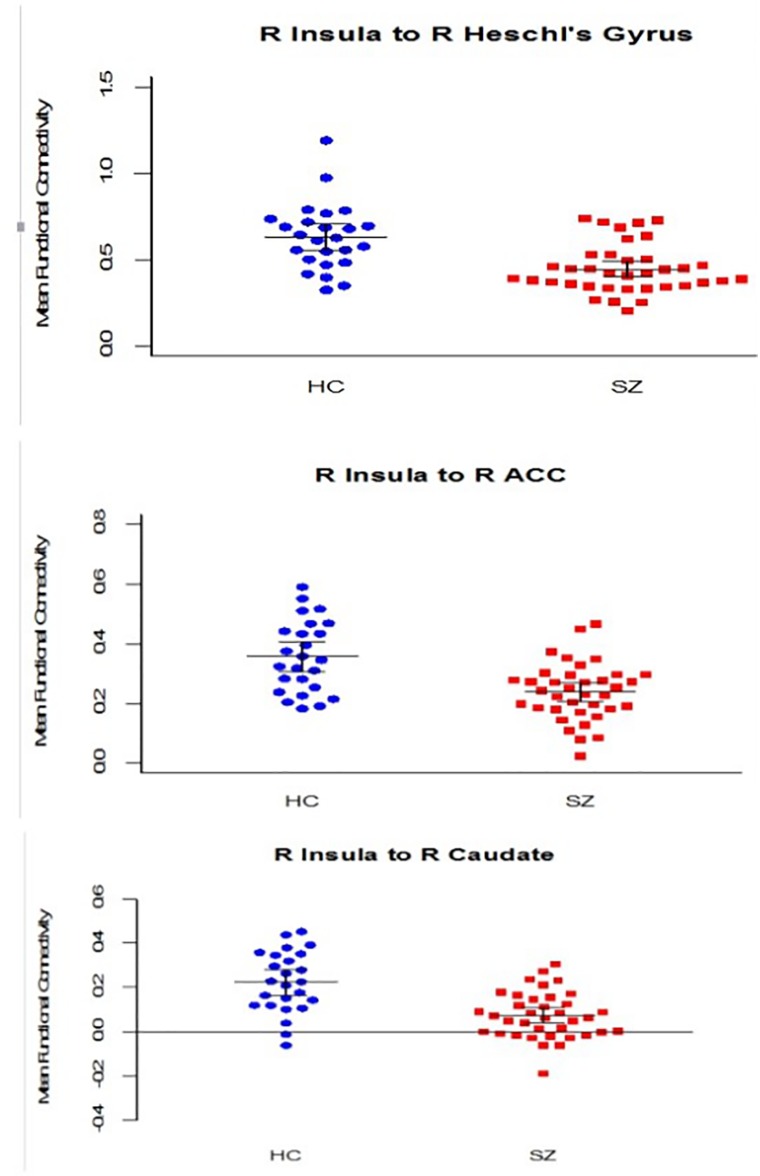
ROI based analysis: group comparisons in the right hemisphere. Note: Regions of right insula connectivity with significant group difference (p < 0.001, with Bonferroni corrections): right anterior cingulate, right Heschl’s gyrus and right caudate. The horizontal lines represent means and the error bars represent 95% confidence intervals.

S1 Table shows the complete results of the seed-based analysis for each hemisphere. In the left hemisphere, no regions met the p <.001 criterion (with Bonferroni corrections) for between-group differences. However, seven regions including caudate, Heschl’s gyrus, posterior cingulate, hippocampus, anterior and posterior parahippocampal gyrus and central operculum showed a trend-level difference (p’s <.05). In the right hemisphere, three regions met the significance criterion (Heschl’s gyrus, anterior cingulate and caudate), and five regions showed a trend-level difference (central operculum, putamen, planum polare, planum temporale and thalamus). Fig 1 shows the group differences in average connectivity for the regions of right insular cortical connectivity that had significant group difference. The regions include the right insular cortical connectivity to the right anterior cingulate cortex (ACC), right Heschl’s gyrus, and right caudate nucleus. Each of these regions showed a significant decrease in the SZ group compared with the HC group.

### 3.2. Confirmatory voxel-based analysis

**Fig 2:**
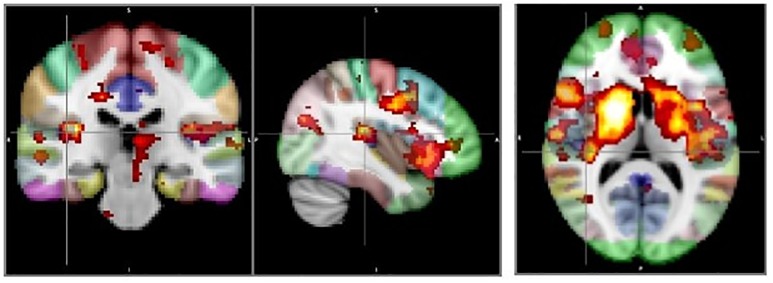
Voxel-based analysis for the right hemisphere insula-seeded correlation maps. Note: FDR-corrected significant clusters: Heschl’s gyrus, superior temporal gyrus (p’s < 0.05); FDR trend-level clusters: anterior cingulate gyrus, caudate.

Fig 2 shows the results of the VBM analysis for the right hemisphere insula-seeded correlation maps. A FDR—corrected significant cluster was observed in Heschl’s gyrus. An additional significant cluster was observed in the superior temporal gyrus. FDR trend-level clusters were also observed in the anterior cingulate gyrus and caudate nucleus. Thus, of the three ROI-based regions in the right hemisphere with significant betwee-group difference, only the Heschl’s gyrus difference was confirmed in the VBM analysis.

### 3.3. Correlation with clinical symptoms

**Fig 3:**
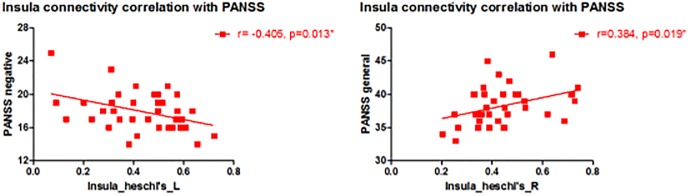
Relationship between insula functional connectivity and clinical symptoms.

The relationship between the resting state average Z-scores of insula-Heschl’s gyrus connectivity and the measures of clinical symptoms were examined in both hemispheres. As shown in Fig 3, there was a significant negative correlation between the left insula-Heschl’s gyrus connectivity and the PANSS negative symptoms subscale scores (r = -0.406, p = 0.019), and a significant positive correlation between the right insula—Heschl’s gyrus and the PANSS general psychopathology subscale scores (r = 0.384, p = 0.019).

## 4. Discussion

Minimizing the potential confounding effect of antipsychotics, the present study provides new evidence regarding changes in insula-Heschl gyrus connectivity in drug naïve, first episode schizophrenia patients. In this study, we found that there was significant reduction in the insular cortical connectivity with Heschl’s gyrus, anterior cingulate cortex (ACC), and caudate nucleus in the right hemisphere. We also found that the insula-Heschis connectivity might be associated withclinical symptoms of schizophrenia.

Reduced insular cortical connectivity with Heschl’s gyrus in the right brain hemisphere was found in the seed-based analysis and was confirmed in the VBM analysis. Similar to insular cortex, Heschl's gyrus (transverse temporal gyrus) may also play an important role in the neural mechanism of schizophrenia[7]. It has been reported that first-episode schizophrenia patients have bilateral Heschl’s gyrus gray matter volume reduction[28], and decreased left Heschl’s gyrus gray matter volume over time [29,30]. Heschl's gyrus contains the primary auditory cortex (PAC), which is the first cortical structure to process incoming auditory information[7]. The PAC is in close spatial proximity to the posteroinferior insular cortex [7]. The auditory information related to prosodic processing may be conveyed to the posterior insular cortex directly through links with the primary auditory and association areas [31]. These findings suggest that deficits in the right insula-Heschl’s functional connectivity may be engaged in the prosody dysfunction in patients with schizophrenia.

Reduced right insula-caudate connectivity is another finding in the present study based on the seed-based analysis. Caudate nucleus is related to successful goal-directed action [32]. Studies have found that schizophrenia and their relatives have a reduced caudate volume [33,34] and compromised white matter integrity[35]. Using functional magnetic resonance imaging to study brain activation during a Monetary Incentive Delay task, Mucci et al. [36] found that avolition in patients with schizophrenia might be related to dorsal caudate hypo-activation. Another research team has reported an abnormal hemispheric specialization of caudate nucleus connectivity in schizophrenia [37]. Reduced insula-caudate connectivity found in our study may play an important role in the development of schizophrenia.

We also found significantly decreased right insula connectivity with ACC using the ROI-based analysis. The insula and ACC together constitute the SN, an intrinsic large-scale network showing strong functional connectivity [38,39]. Menon and Uddin [11] have recently proposed that the primary role of this network is to segregate the most relevant stimuli among internal and extra-personal stimuli, to assist target brain regions and generate appropriate behavioral responses to salient stimuli, and to enable switching between the default mode and task-related states of brain connectivity. Abnormal connectivity of SN has been reported to be associated with the negative symptoms, hallucinations, delusions and other psychotic symptoms of schizophrenia[40–44]. Previous studies have suggested that functional and/or structural alterations within the insular cortex might contribute to aberrant salience processing, leading to the development of schizophrenia symptoms[7].

In the present study, our correlation analysis within the patient group showed a significant negative relationship between left insula-Heschl’s gyrus connectivity and the PANSS negative symptoms subscale scores, suggesting the impairment in this particular functional connectivity might be related to the development of negative symptoms of schizophrenia. In addition, the correlation analysis found a significant positive relationship between the right insula-Heschl gyrus connectivity and the PANSS general psychopathology subscale scores, suggesting that an abnormally increased connectivity might contribute to the manifestation of clinical symptoms such as depression and anxiety observed in patients with schizophrenia. Symptoms of schizophrenia have been attributed to a failure of functional integration or aberrant connectivity among regions or systems of the brain [45]. Manoliu et al. found that the right anterior insular cortical dysfunction was associated with positive symptoms of schizophrenia during the acute phase of psychosis; they further suggested that the specific SN/DMN/CEN reorganization with distinct insular cortical pathways might be related to different symptom domains of schizophrenia [17]. Another study reported that structural alterations of the insular cortex might be related to negative symptoms during psychotic remission, which is consist with our report about the negative correlation between the insular cortex-Heschl’s gyrus connectivity in the left brain hemisphere and the PANSS negative symptoms [16]. Lee et al. reported that progressive gray matter volume reduction in both insular cortex and temporal pole in first episode psychosis was inversely associated with changes in the overall Brief Psychiatric Rating Scale symptom scores [46]. Some studies suggested that disturbances in emotional prosody is due to the impairment in early auditory sensory processing, which may contribute to later impairment in attention-dependent processes in schizophrenia [47]. It is also reported that the cortical surface area and local white matter volume of posterior insula might play an important role in insight impairment in schizophrenia [48]. Both attention deficit and insight impairment might be related to the general psychopathology symptoms (depression, anxiety etc.) observed in patients with schizophrenia.

The present study has several limitations: 1) Some meaningful information may have been lost by removing global signal in preprocessing of the imaging data. 2) The patient subjects were recruited from an inpatient unit; the overall symptom severity of the study sample might be higher than patients in the outpatient setting. Therefore the findings from this study, especially the correlation between functional connectivity and clinical symptoms, may not be generalizable to all patients. 3) The correlation analysis between functional connectivity and clinical symptoms was not corrected for multiple correlations; 4) Several software tools were used for pre-processing and processing of imaging data; 5) The PANSS was the only rating scale used to assess clinical symptoms.

In summary, our study demonstrates the abnormal functional connectivity of insula, insula-Heschl gyrus connectivity in particular, in drug naïve, first episode schizophrenia patients. These abnormalities might contribute to the development of schizophrenia. Future studies with a larger sample size and in combination with other techniques such as diffusion tensor imaging (DTI) are needed to further assess the regions of insular connectivity in relation to the development and treatment of schizophrenia.

## Supporting Information

S1 Table The complete results of group comparison in connectivity values among the insular cortex with ipsilateral cortical ROIs. (DOC)
