# Supplementary material for: Decreased Functional Connectivity of Insular Cortex in Drug Naïve First Episode Schizophrenia: In Relation to Symptom Severity
Source: PLoS One. 2017 Jan 20;12(1):e0167242. doi: 10.1371/journal.pone.0167242 (PMC5249106; doi:10.1371/journal.pone.0167242)
Supplement: S1 Table — (DOC) [file pone.0167242.s001.doc]

**Table 2 (supplementary)**

**The complete results of group comparison in connectivity values among the insular cortex with ipsilateral cortical ROIs**

| Seed ROI = Right Insular Cortex  Ipsilateral (Right) Target ROI | Mean_HC | Mean_SZ | T test p value |
| --- | --- | --- | --- |
| Heschl's Gyrus - includes H1 and H2  Cingulate Gyrus - anterior division  Caudate  Central Opercular Cortex  Putamen  Planum Polare  Planum Temporale  Thalamus-Proper  Cingulate Gyrus - posterior division  Paracingulate Gyrus  Middle Temporal Gyrus - posterior division  Inferior Temporal Gyrus - posterior division  Frontal Orbital Cortex  Inferior Frontal Gyrus - pars opercularis  Superior Temporal Gyrus - posterior division  Superior Frontal Gyrus  Precuneous Cortex  Superior Parietal Lobule  Frontal Operculum Cortex  Middle Frontal Gyrus  Temporal Fusiform Cortex - posterior division  Supracalcarine Cortex  Middle Temporal Gyrus - temporooccipital part  Parietal Operculum Cortex  Precentral Gyrus  Amygdala  Intracalcarine Cortex  Inferior Temporal Gyrus - temporooccipital part  Juxtapositional Lobule Cortex - formerly Supplementary Motor Cortex  Cuneal Cortex  Parahippocampal Gyrus - posterior division  Parahippocampal Gyrus - posterior division  Frontal Pole  Supramarginal Gyrus - anterior division  Temporal Pole  Accumbens area  Inferior Frontal Gyrus - pars triangularis  Lingual Gyrus  Lateral Occipital Cortex - inferior division  Superior Temporal Gyrus - anterior division  Temporal Fusiform Cortex - anterior division  Angular Gyrus  Frontal Medial Cortex  Occipital Fusiform Gyrus  Supramarginal Gyrus - posterior division  Lateral Occipital Cortex - superior division  Postcentral Gyrus  Occipital Pole  Inferior Temporal Gyrus - anterior division  Hippocampus  Temporal Occipital Fusiform Cortex  Subcallosal Cortex  Pallidum  Middle Temporal Gyrus - anterior division | 0.6236  0.3777  0.2346  0.6163  0.5105  0.6146  0.5107  0.2580  0.0790  0.1685  0.0330  -0.1048  0.2883  0.3101  0.3262  0.0324  -0.0504  0.0483  0.6294  0.0109  -0.0368  -0.0248  0.1000  0.3964  0.1881  0.2907  -0.0076  -0.0285  0.2259  -0.0411  0.1618  0.1618  0.0201  0.3108  0.1189  0.2189  0.1803  0.0086  -0.0409  0.3303  0.0537  0.0191  0.0177  0.0103  0.2264  -0.0907  0.1269  -0.0742  -0.0204  0.1374  0.0571  0.0661  0.2979  -0.0499 | 0.4293  0.2615  0.1033  0.5050  0.3776  0.5074  0.3989  0.1836  0.0166  0.1020  -0.0268  -0.0614  0.2260  0.2517  0.2711  -0.0030  -0.0865  0.1007  0.5509  -0.0285  0.0231  -0.0719  0.0574  0.3503  0.1622  0.2501  -0.0437  0.0040  0.1923  -0.0673  0.1320  0.1320  0.0040  0.2809  0.1024  0.1804  0.1587  -0.0098  -0.0587  0.3098  0.0399  0.0027  0.0047  -0.0022  0.2394  -0.0841  0.1223  -0.0785  -0.0250  0.1414  0.0539  0.0689  0.2988  -0.0498 | 0.0001  0.0005  0.0009  0.0024  0.0074  0.0081  0.0194  0.0309  0.0554  0.0755  0.0836  0.1244  0.1432  0.1454  0.1479  0.1756  0.1759  0.1791  0.1824  0.1964  0.2055  0.2328  0.2455  0.3228  0.3307  0.3473  0.3516  0.4346  0.4589  0.4803  0.4805  0.4805  0.5151  0.5357  0.5409  0.5801  0.5897  0.5917  0.5917  0.6361  0.6785  0.6981  0.7136  0.7399  0.7575  0.8094  0.8705  0.8706  0.9125  0.9153  0.9284  0.9462  0.9884  0.9990 |
